# RTB Lectin: a novel receptor-independent delivery system for lysosomal enzyme replacement therapies

**DOI:** 10.1038/srep14144

**Published:** 2015-09-18

**Authors:** Walter Acosta, Jorge Ayala, Maureen C. Dolan, Carole L. Cramer

**Affiliations:** 1 Arkansas Biosciences Institute at Arkansas State University-Jonesboro, State University, Arkansas, USA; 2 Department of Biological Sciences, Arkansas State University-Jonesboro, State University, Arkansas, USA; 3 BioStrategies LC, State University, Arkansas, USA

## Abstract

Enzyme replacement therapies have revolutionized patient treatment for multiple rare lysosomal storage diseases but show limited effectiveness for addressing pathologies in “hard-to-treat” organs and tissues including brain and bone. Here we investigate the plant lectin RTB as a novel carrier for human lysosomal enzymes. RTB enters mammalian cells by multiple mechanisms including both adsorptive-mediated and receptor-mediated endocytosis, and thus provides access to a broader array of organs and cells. Fusion proteins comprised of RTB and human α-L-iduronidase, the corrective enzyme for Mucopolysaccharidosis type I, were produced using a tobacco-based expression system. Fusion products retained both lectin selectivity and enzyme activity, were efficiently endocytosed into human fibroblasts, and corrected the disease phenotype of mucopolysaccharidosis patient fibroblasts *in vitro*. RTB-mediated delivery was independent of high-mannose and mannose-6-phosphate receptors, which are exploited for delivery of currently approved lysosomal enzyme therapeutics. Thus, the RTB carrier may support distinct *in vivo* pharmacodynamics with potential to address hard-to-treat tissues.

Lysosomal storage diseases (LSDs) represent a group of rare genetic disorders involving deficiencies of specific lysosomal enzymes, leading to the pathological accumulation of undegraded substrates in cells, tissues, and organs of affected patients. Enzyme replacement therapy (ERT) remains the hallmark treatment approach for LSDs and is based on regular, intravenous administration of recombinant lysosomal enzymes. All currently approved ERTs exploit glycan structures present on the replacement enzyme that mediate cell uptake based on interactions with high-mannose or mannose-6-phosphate receptors. These ERT strategies have been effective in correcting many of the symptoms associated with somatic organs and have significantly enhanced patient quality of life. However, some organs and tissues associated with these diseases are not effectively addressed with current ERTs^1^,^2^. These “hard-to-treat” tissues include most notably the brain and other tissues of the central nervous systems, as well as lung, heart, bone and cartilage. There is significant interest in identifying new ERT delivery strategies that exploit different uptake mechanisms in facilitating delivery to a broader array of cells and tissues.

Our research has focused on development of the plant lectin RTB as a new protein carrier for lysosomal ERT delivery. RTB is the nontoxic carbohydrate-binding B subunit of ricin AB toxin from *Ricinus communis*. In nature, RTB directs uptake and trafficking of the ribosome-inactivating toxin A subunit (RTA) of ricin in mammalian cells. RTB facilitates uptake by targeting cell surface glycoproteins and glycolipids with β-1,4-linked galactose or galactosamine residues abundant on mammalian cells^3^. Based on inhibitor studies, co-trafficking analyses with compartment-specific markers, and use of mutant cell lines depleted of specific pathways, RTB-directed ricin has been reported to enter cells by at least six distinct endocytic routes^4^,^5^,^6^. These include endocytosis via clathrin-dependent mechanisms, and clathrin-independent mechanisms (which account for up to 70% of fluid uptake in some cells^4^), including pinocytosis/macropinocytosis^7^. The dominant route of RTB-mediated uptake involves absorptive-mediated rather than receptor-mediated mechanisms^8^. As all cell types tested are sensitive to ricin, albeit at different levels^3^, we propose that the nontoxic RTB lectin may be an effective carrier for delivering large glycoprotein therapeutics into a broad array of cells with significant potential to enhance disease correction in tissues that are not effectively addressed by current ERTs.

As a first step in determining whether RTB can deliver corrective doses of a human ERT into disease cells and lysosomes, we produced RTB genetic fusions with human α-L-iduronidase (IDUA). IDUA is the replacement enzyme for mucopolysaccharidosis type I (MPS I, also called Hurler, Hurler-Scheie Syndrome) and is responsible for degrading glycosaminoglycans (GAG; e.g., heparan sulfate and dermatan sulfate). Severe IDUA deficiency (Hurler) leads to profound visceral, skeletal, cardiac and neurological abnormalities and early death. An FDA-approved ERT for MPS I was approved in 2003, but has not been effective in addressing the CNS-related pathologies^1^,^2^. Here we describe the production of human IDUA and RTB:IDUA fusions in the leaves of intact *Nicotiana benthamiana* plants using a transient expression system. The purified RTB:IDUA fusion product retained both lectin-binding and IDUA enzymatic activities, was efficiently taken up by human cells, and reduced GAG levels in Hurler patient fibroblasts.

## Results

### Expression of IDUA and RTB:IDUA in *N. benthamiana* leaves

**Figure 1:**
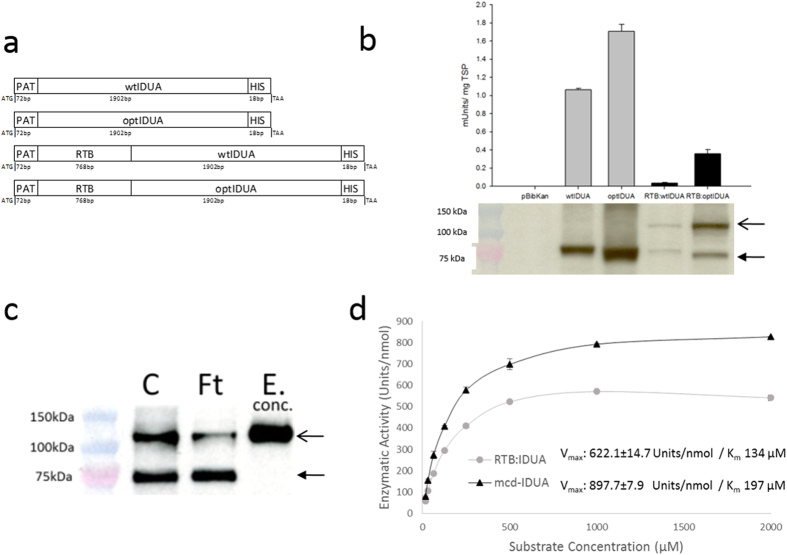
Production of IDUA and RTB:IDUA fusions in plants. (**a**) Construct design of the recombinant proteins used for plant-based expression. PAT, plant signal peptide from potato patatin gene; RTB, RTB lectin; wtIDUA, native human alpha-L iduronidase; optIDUA, codon-optimized IDUA; HIS, 6X histidine tag. (**b**) IDUA yields in crude leaf extracts as assessed by IDUA enzyme activity and Western immunoblot. Analyses represent data from at least four biological replicates comparing extracts of *N. benthamiana* leaves harvested at the peak time-point for each construct: wtIDUA, 48 h; optIDUA, 120 h; RTB:wtIDUA, 72 h; RTB:optIDUA, 72 h; and pBibKan “empty vector” control at 72 h. For the Western blot, 40 μg total soluble protein/lane were size-separated (SDS-PAGE), transferred to nitrocellulose membranes and detected using an anti-IDUA antibody. (**c**) Lectin binding activity of fusion protein. Western blot analysis of key fractions before and after lactose affinity chromatography; fractions were detected using anti-IDUA antibodies: crude extract (C), flow-through fraction (Ft), and concentrated elution fraction (E-conc; concentrated 10-fold). Arrows in (**b**) and (**c**) approximate the expected sizes for full-length RTB:IDUA (open arrow; ~110 kDa) and for IDUA (closed arrow; ~75  kDa). (**d**) Michaelis-Menten enzyme kinetics of mcd-IDUA and RTB:IDUA purified extracts. IDUA enzyme activity was determined for various concentrations of IDUA using mammalian cell-derived IDUA (mcd-IDUA; certified as >99% purity (R&D System)) and plant-derived RTB:IDUA (purified by lactose affinity and size exclusion chromatography yielding a product of 98% purity; see Supplementary Fig. S1 online).

Human IDUA has been previously produced in transgenic plants or plant cells, including *Nicotiana* species^9^,^10^,^11^,^12^. In all cases, plant-made IDUA was enzymatically active but yields were relatively low. We therefore tested two approaches for increasing IDUA product yield: codon optimization and transient expression strategies. As the native human IDUA coding sequence (wtIDUA) is very GC-rich (67%), the gene was re-synthesized based on tobacco codon preferences yielding a 44% GC sequence (optIDUA). Plant expression constructs containing wtIDUA or optIDUA, with or without an N-terminal RTB fusion partner, were developed as diagrammed in Fig. 1a. These constructs were used for *Agrobacterium*-mediated transient expression in leaves of intact *N. benthamiana* plants.

The harvest time of infiltrated leaves that support greatest product yield is protein-dependent^13^. Therefore, initial studies compared product yields by Western immunoblot analysis of proteins from leaves harvested at 48, 72, 96, and 120 h after *Agrobacterium* infiltration of each construct. Subsequent comparisons utilized the leaves harvested at the highest yielding time-point for each construct. Western analyses showed cross-reactive product of the predicted sizes (approximately 75 kDa for IDUA and 110 kDa for the RTB:IDUA fusion protein) detected with an anti-IDUA monoclonal antibody (Fig. 1b). In contrast, extracts from leaves infiltrated with *Agrobacterium* bearing an “empty vector” (pBibKan) showed no cross-reactive proteins or IDUA activity. Yields of the fusion products were lower than IDUA alone. Codon-optimized versions of IDUA (optIDUA and RTB:optIDUA) provided substantial yield improvements (Fig. 1b) and were selected for all further analyses, subsequently referred to as plant-derived IDUA (pld-IDUA) and RTB:IDUA.

### Characterization of RTB:IDUA fusion protein

The IDUA enzymatic and lectin-binding activities of plant-made RTB:IDUA were evaluated. Crude extracts of pld-IDUA and RTB:IDUA showed IDUA activity (Fig. 1b) indicating that the C-terminal HIS-tag and N-terminal RTB did not eliminate enzymatic function. Western analyses of the RTB:IDUA fusions revealed a breakdown product (~75 kDa) that cross-reacted with anti-IDUA antibodies, which suggests there may be a cleavage-sensitive site between the lectin and enzyme domains. To confirm that IDUA activity was associated with RTB:IDUA and not due solely to the 75 kDa product (presumably IDUA that has lost RTB), RTB:IDUA was further enriched using lectin affinity chromatography (Fig. 1c). The lactose-affinity step yielded an RTB:IDUA-enriched fraction that lacked the IDUA breakdown product but retained both lectin and IDUA enzyme activity.

In order to selectively detect and quantify only lectin-active/IDUA-active RTB:IDUA in crude extracts and in purified fractions, a dual bioactivity assay was designed as a modification of the asialofetuin “functional ELISA” used to quantify RTB lectin binding. In this assay, the galactose-rich asialofetuin glycoprotein serves as the capture molecule for RTB, with a 4-MUI-based fluorescence assay subsequently used to quantify IDUA activity in lectin-active product. Comparing total IDUA activity in crude extracts with asialofetuin-bound IDUA activity (i.e. lectin-active RTB:IDUA), the asialofetuin-bound IDUA activity represents only 21% of the total value obtained in the crude plant extracts (0.08 Units/mg TSP compared to 0.4 Units/mg TSP of total IDUA activity). However, a relatively small proportion of RTB:IDUA was detected in the lactose column flow-through compared to crude fractions (Fig. 1c). This discrepancy suggests that the RTB fusion partner may reduce the specific activity of IDUA. To directly assess this, enzyme kinetics of purified RTB:IDUA (using lactose and size exclusion chromatography) were compared with mammalian cell-derived IDUA (mcd-IDUA). The identity of purified RTB:IDUA was further confirmed by mass spectrometry of tryptic peptides with 32 exclusive unique peptides identified (7 specific for RTB including the N-terminal peptide and 25 for IDUA) and representing 37% coverage (see Methods and Supplementary Fig. S2 online). Molar specific activities of the purified enzymes were estimated by measuring the Units (nmol of 4MU/min) generated per nmol of protein (Units/nmol). Under our assay conditions, RTB:IDUA has a V_max_ of 622.1 ± 14.7 Units/nmol and a K_m_ of 134 μM, in comparison to mcd-IDUA with a V_max_ of 897.7 ± 7.9 Units/nmol and K_m_ of 197 μM (Fig. 1d). These data suggest that the N-terminal placement of RTB reduces IDUA specific activity, which was observed previously with IDUA bearing an N-terminal RAP (receptor-associated protein) fusion^14^.

### Cellular uptake, GAG reduction and phenotype correction

**Figure 2:**
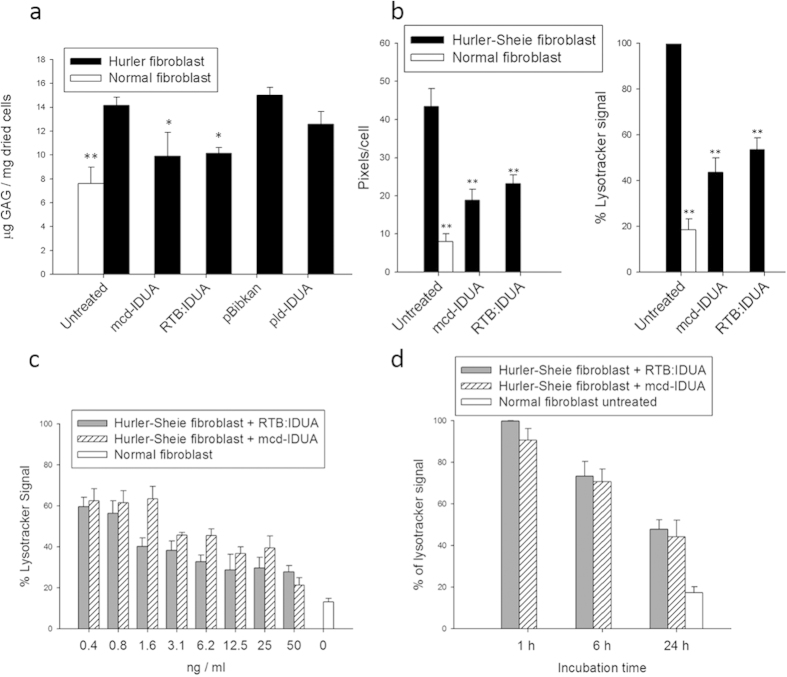
Reduction of GAG substrate and lysosomal volume in Hurler and Hurler-Scheie fibroblasts by RTB:IDUA. (**a**) IDUA uptake and glycosaminoglycan (GAG) correction in Hurler disease fibroblasts. GAG levels were determined in confluent cultures of normal human fibroblasts (white bar) or Hurler patient fibroblasts (black bars) that were untreated or treated for 24 h with IDUA [at 1 Unit (nmol/min) IDUA equivalents per ml of cell culture media] or “empty-vector” control fractions. mcd-IDUA, mammalian cell-derived IDUA; RTB:IDUA, plant-derived fusion protein; pld-IDUA, plant-derived IDUA; pBibKan, similarly processed protein fractions from “empty-vector” leaf extract. Each point is based on at least 3 independent culture plates (see Methods). (**b**) IDUA correction of lysosomal phenotype based on high throughput imaging assay. Untreated normal, untreated Hurler-Sheie fibroblasts and enzyme treated (50 ng/ml culture medium for 24 h) Hurler-Sheie fibroblast were analyzed for LysoTracker® signal as described in Methods. Data was expressed as percentage of LysoTracker® signal detected relative to the total signal obtained from the untreated Hurler-Sheie fibroblast. (**c**) Dose response for RTB:IDUA-mediated correction of Hurler-Sheie cells. Cells were treated for 24 h at the indicated protein concentrations of mcd-IDUA or RTB:IDUA (ng purified protein/ml culture media; not adjusted to IDUA equivalents) and then analyzed based on percentage of LysoTracker® signal per cell. (**d**) Kinetics of phenotype correction comparing mcd-IDUA and RTB:IDUA. Hurler-Sheie cells treated with mcd-IDUA or RTB:IDUA (50 ng/ml culture medium) were stained, fixed and analyzed at times indicated. Statistical significance vs. untreated was assessed by Dunnett’s multiple comparisons test, **P < 0.005, *P < 0.05. All experiments were normalized to untreated Hurler or Hurler-Sheie fibroblasts grown in the same plate and conditions to accommodate for experiment-to-experiment variations in GAG levels and lysosomal phenotype are observed in disease fibroblast lines due passage number and growth rates.

To test whether RTB:IDUA is capable of delivering its enzymatic cargo to the lysosomal site of GAG substrate accumulation, lectin-active/IDUA-active RTB:IDUA was used to treat human fibroblasts from normal and iduronidase-deficient (Hurler and Hurler-Scheie syndrome patients) individuals. In addition to RTB:IDUA, two controls samples were affinity-purified from infiltrated leaves: 1) pld-IDUA, plant-derived IDUA (purified based on the His-tag), and 2) pBIB-Kan, the plant-background negative control comprised of equivalent lactose affinity-purified fractions from leaves infiltrated with *A. tumefaciens* bearing a pBIB-Kan “empty vector”. The final IDUA-containing products were quantified based on IDUA enzymatic activity and, unless noted, treatments of human fibroblasts were based on equivalent IDUA activity units. As shown in Fig. 2a, treatment of Hurler cells for 24 h with RTB:IDUA resulted in significant reduction in intracellular GAG content (p < 0.05) with correction equivalent to that observed with mammalian cell-derived IDUA (mcd-IDUA). In contrast, treatment of cells with “empty vector” control plant fractions (pBIB-Kan) did not reduce GAG content, indicating that no endogenous components from plants impacted GAG levels in fibroblasts. Some GAG reduction was seen in the Hurler cells treated with plant-derived IDUA. However, these levels were not significantly different when compared to the controls. It should be noted that plant-made IDUA will contain mannose-terminated glycans and thus, a low levels of uptake may be possible via high mannose receptor interactions, although this route is clearly less efficient than the adsorptive-mediated uptake conferred by RTB. Analogous experiments were subsequently performed using a Hurler-Scheie patient fibroblast line. Hurler-Sheie fibroblasts showed a higher GAG accumulation upon reaching confluence than the Hurler cell line and treatment with RTB:IDUA or mcd-IDUA substantially reduced GAG levels. These results indicate that the RTB carrier delivers corrective levels of active IDUA to the intracellular sites of GAG accumulation.

RTB:IDUA-mediated correction of the MPS I cellular disease phenotype was further characterized using a newly developed high-throughput imaging assay that captures lysosome area and number based on staining with LysoTracker^®^ red (see Methods). Image data from treated or untreated cells were analyzed based on pixels/cell (total LysoTracker^®^red pixels per image divided by cell number based on DAPI-stained nuclei) under fixed magnification and capture parameters. This assay effectively distinguished normal and Hurler-Scheie fibroblasts, with Hurler-Scheie fibroblasts showing significantly higher lysosomal signal (80% higher red pixels/cell) consistent with extensive GAG accumulation (Fig. 2b). Treatment of Hurler-Scheie fibroblasts with 50 ng/ml of the IDUA-containing recombinant proteins for 24 h, resulted in signal reduction from 100% to about 40% (Fig. 2b). This method was used to compare the kinetics of lysosomal phenotype corrections of mcd-IDUA and RTB:IDUA under varying doses (Fig. 2c) and times of treatment (Fig. 2d). Following 24 h incubation with different concentrations of recombinant protein, RTB:IDUA demonstrated similar efficacy for phenotype correction of the Hurler-Sheie fibroblast compared to mcd-IDUA (Fig. 2c). It should be noted that equivalent amounts (ng) of purified protein were used in this assay as opposed to molar equivalents, suggesting that RTB:IDUA may actually be more effective than mcd-IDUA under these conditions even with the lower apparent molar V_max_. The kinetics of lysosomal correction (Fig. 2d) were similar for Hurler-Sheie fibroblasts treated with either RTB:IDUA or mcd-IDUA (50 ng/ml).

### RTB:IDUA uptake is independent of high mannose receptor - and mannose-6-phosphate receptor-mediated routes

**Figure 3:**
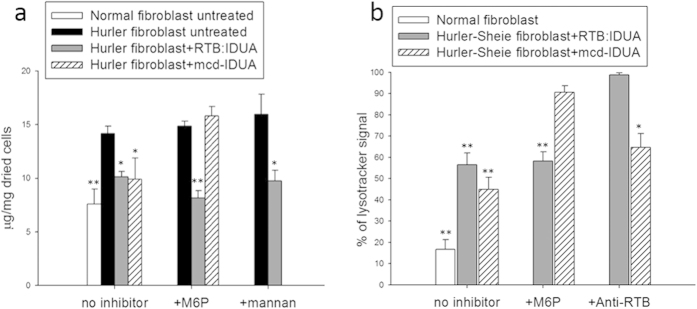
RTB:IDUA-mediated correction is independent of MMR and M6PR. (**a**) Impact of M6PR- and MMR-competitive inhibitors on GAG reduction in treated Hurler fibroblasts. Fibroblast cells were pre-incubated with 4 mM D-mannose-6-phosphate sodium salt or 4 mg/ml mannan (yeast-derived) for two hours prior to treatment with 1 Unit/ml culture medium of RTB:IDUA or mcd-IDUA. As controls, untreated normal and Hurler fibroblasts were incubated for 24 hours in serum-free MEM or serum-free MEM containing 4 mM M6P or 4 mg/ml mannan. Intracellular GAG content was measured as described in Methods. Data for each treatment is based on 3 independent cultures, pooled and weighed in fractions (from 0.8–1.5 mg) with a minimum of 4 fractions per treatment analyzed. (**b**) Impact of M6P and RTB antibodies on LysoTracker® phenotype in treated Hurler fibroblasts. Fibroblast cells were pre-incubated with 4 mM D-mannose-6-phosphate sodium salt two hours prior to treatment with 50 ng/ml culture medium of RTB:IDUA or mcd-IDUA. Untreated normal and Hurler fibroblast mock controls were incubated for 24 h in serum-free MEM or serum-free MEM containing 4 mM M6P. For RTB antibody treatments, RTB:IDUA and mcd-IDUA proteins were pre-incubated with RTB antibody for 2 h prior to addition to the cells. Comparison of enzyme treatments were assessed at 24 h using the LysoTracker® high-throughput assay outlined in Fig. 2 with statistical significance of treated vs. untreated samples assessed by Dunnett’s multiple comparisons test (**P < 0.005, *P < 0.05).

We propose that RTB delivers lysosomal enzymes by fundamentally different mechanisms than currently approved lysosomal ERTs which exploit either high-mannose (MMR) or mannose-6-phosphate (M6PR) receptors to gain access to cells and lysosomes. Unlike mammalian cells, plant cells do not use mannose-6-phosphate-terminated glycans to direct enzymes to vacuoles (plant lysosomal compartment) and do not possess the enzymatic machinery to make this modification^15^. Thus, the results described above suggest that RTB:IDUA accesses cells and lysosomes by M6PR-independent routes. The N-linked glycans of plant-made glycoproteins include mannose-terminated forms which could contribute to uptake of RTB:IDUA and plant IDUA by a high mannose receptor (MMR) route^16^,^17^. In order to assess a potential role of either M6PR or MMR in the internalization of plant-derived RTB:IDUA, uptake assays were performed in the presence of the respective competitive inhibitors, M6P sodium salt and mannan (Fig. 3a). Saturation of the receptors was achieved by incubating the cell cultures for two hours prior to enzyme treatment with doses of inhibitors reported to block 95% of uptake^18^. Cells were subsequently treated with the various IDUA enzymes for 24 h. While the presence of mannose-6-phosphate inhibitor clearly blocked the ability of mcd-IDUA to reduce GAG substrate levels in Hurler fibroblasts, the inhibitor had no significant effect on RTB:IDUA’s ability to reduce GAG substrate content in cells (P < 0.005). We also tested RTB:IDUA-mediated uptake and GAG reduction in the presence of inhibitory levels of mannan to determine if RTB-mediated intracellular delivery was based on MMR interactions. As shown in Fig. 3a, RTB:IDUA in the presence of mannan effectively reduced GAG substrate in Hurler fibroblasts to levels similar to those attained from cells treated with either mcd-IDUA or RTB:IDUA in the absence of inhibitor (P < 0.005).

We corroborated M6P-independent uptake by measuring phenotypical changes in the cell lines using the high-throughput imaging assay described above. In contrast to that observed in the absence of inhibitors, mcd-IDUA was unable to reduce the LysoTracker^®^ signal in Hurler-Sheie fibroblast when M6P receptors were blocked (Fig. 3b). However, M6P receptor saturation did not impede the ability of RTB:IDUA to reduce the LysoTracker^®^ signal in disease fibroblasts to levels obtained in the absence of inhibitors. To further characterize the role of RTB lectin activity in the uptake of IDUA, polyclonal antibodies to RTB (shown to be partially neutralizing; Liu, 2006, etd-12202006-220049) were used to block the sugar binding activity of RTB. Hurler-Sheie fibroblasts were treated with RTB:IDUA that had been pre-incubated with anti-RTB antibodies. These cells showed no reduction in LysoTracker^®^ signal (i.e., no lysosomal phenotype correction) indicating that uptake and/or trafficking of RTB:IDUA was blocked (Fig. 3b). In contrast, anti-RTB antibodies did not prevent mcd-IDUA-mediated correction of the lysosomal phenotype of disease fibroblasts. These results suggest that the dominant route(s) of RTB-mediated uptake is independent of either M6PR or MMR but is dependent on sugar-binding and lectin-mediated endocytosis.

## Discussion

In summary, we have demonstrated three main points. First, plants are capable of producing large bioactive glycoprotein fusion products comprising an RTB lectin and human lysosomal enzyme that retain both lectin selectivity and α-L-iduronidase enzyme activity. Second, the RTB:IDUA fusion product effectively reduces the GAG disease substrate and corrects the lysosomal phenotype of Hurler and Hurler-Scheie patient fibroblasts with kinetics equivalent to mammalian-cell-derived IDUA. This data represents the first demonstration that the plant lectin RTB delivers fully functional enzymes into cells and into the critical subcellular sites of lysosomal disease substrate accumulation. Third, RTB:IDUA corrects the disease phenotype of Hurler and Hurler-Scheie fibroblasts by mechanisms that are independent of either high-mannose receptors (MMR) and M6P receptors suggesting the potential for distinct *in vivo* pharmacodynamics compared to current ERTs.

All current FDA-approved LSD enzyme replacement therapeutics rely on one of only two mechanisms to carry corrective enzyme into cells and to the lysosomal sites of disease substrate accumulation. ERTs for Gaucher disease utilize glucocerebrosidase modified to display terminal mannose residues on their N-linked glycans. This modification directs cell uptake via the high mannose receptors (MMR), which are particularly abundant on macrophages and other cells of monocyte lineage - cells that show significant disease involvement in Gaucher patients. Currently approved ERTs for Fabry, Pompe and mucopolysaccharidosis types I, II, IV A and VI diseases use recombinant enzymes that display N-linked glycans with terminal mannose-6-phosphate residues targeting delivery based on interactions with the M6P receptors. M6P receptors function primarily within the endomembrane system to facilitate delivery of acid hydrolases to the lysosome, especially in mediating trans-Golgi to early/late endosome transport^19^,^20^. M6P-mediated ERT uptake into cells exploits the M6PR salvage pathway that recovers M6PR from the plasma membrane. Although ERTs have been highly beneficial to patients with these diseases, the ineffective correction of neurological, ocular and skeletal disease manifestations remains a major drawback of current therapies dependent upon MMR- and M6PR-dependent delivery. M6P transport to the brain has been demonstrated to be developmentally regulated in early postnatal life and lost in adulthood^21^. Thus, there is keen interest in developing new enzyme delivery strategies with potential to more effectively target a broader array of cells and tissues.

In the current study, we showed that RTB effectively delivers associated human lysosomal enzymes into cells and lysosomes by mechanisms that are independent of either MMR or M6PR. Treatment of Hurler and Hurler-Scheie fibroblasts with RTB:IDUA resulted in reduction of cellular GAG levels to “normal” levels at doses and with correction kinetics equivalent to mammalian cell-derived IDUA. The dominant routes of RTB-mediated uptake into mammalian cells involved absorptive-mediated endocytosis based on lectin interactions with the galactose/galactosamine-containing glycoproteins and glycolipids abundant on mammalian cell surfaces^22^,^23^. In contrast to receptor-mediated uptake, adsorptive-mediated endocytosis is considered less-saturable due to the wider interaction with non-specific moieties expressed at the luminal surface of cells^24^. Using these mechanisms, various plant lectins have been shown to facilitate drug delivery to many of the “hard-to-treat” organs (e.g., eye, lung and brain^25^,^26^, reviewed in^27^). Historically, RTB-mediated uptake based on ricin cytotoxicity has been demonstrated in essentially all of the tissues/cell types considered challenging for therapeutic glycoproteins delivery including peripheral and central neurons, brain, bone, lung and heart^28^,^29^,^30^. We therefore anticipate that RTB will provide greater enzyme delivery to these challenging tissues than ERTs that rely exclusively on MMR and M6PR interactions.

We produced RTB:IDUA using an A*grobacterium*-mediated transient expression system in the leaves of *Nicotiana benthamiana*. Production of the non-toxic RTB lectin in a heterologous plant system eliminates any possible ricin toxicity (no RTA is present). In addition, plant-based production systems may provide advantages in safety (do not support human viruses), cost (especially initial capitalization costs), and scalability compared to mammalian-cell-based systems^31^,^32^. The ability of plant systems to provide clinically efficacious ERTs was demonstrated with FDA approval of glucocerebrosidase (Elelyso) produced in carrot cells for treatment of Gaucher disease. Clinical trials showed no difference in effectiveness or immunogenicity between plant- and animal-cell-derived products^33^,^34^. The plant-based Gaucher ERT has been safely administered to patients since 2007^35^, reducing therapy cost and addressing safety and supply shortfalls with the current CHO-based product. Although glycoproteins made using the *N. benthamiana* platform have not yet attained FDA approval for human use, monoclonal antibodies (e.g., ZMapp antibodies used to treat Ebola) and virus-like particles made using this system are in clinical evaluation and show efficacy in primates and humans^36^,^37^. That plants correctly synthesize and assemble fully functional antibodies underscores their potential to produce highly complex glycoproteins.

In this study, we produced a 110 kDa fusion protein that placed the RTB plant lectin (34 kDa) at the N-terminus of human IDUA (~75 kDa). Active IDUA had been produced previously in plants^9^,^10^,^11^ but at relative low levels. We found that using a “codon-optimized” version of IDUA based on tobacco codon preferences provided greater yields in plants, especially of the large RTB:IDUA fusion (Fig. 1b). Enzyme kinetic analyses comparing RTB:IDUA to mammalian cell-derived IDUA showed a lower V_max_ for the plant-derived fusion proteins (based on molar equivalents) although the enzymes behaved equivalently in the disease correction assays with MPS I fibroblasts (Fig. 2c). It may be that placement of the lectin partner at the N-terminus of IDUA negatively impacts IDUA conformation or substrate access. Consistent with this interpretation, previous studies showed that addition of an unrelated peptide to the N-terminus of IDUA also resulted in reduced IDUA enzyme kinetics^14^. To further test impacts of fusion orientation, we recently produced fusions that placed RTB at the C-terminus of IDUA (IDUA:RTB). Preliminary analyses indicate that this IDUA:RTB fusion provided IDUA activity and kinetic values equivalent to mcd-IDUA (Condori, Acosta and Cramer, unpublished results).

Lysosomal storage diseases represent a devastating group of rare diseases and ERT therapies have revolutionized patient care in those diseases where they are available. New delivery strategies are needed to address the complex clinical presentations of these diseases and to mobilize corrective enzyme to sites such as brain and bone that are not treated by the current generation of ERTs. The research presented here introduces a new ERT carrier system based on the plant RTB lectin that shows efficient delivery of corrective enzyme to disease cells and lysosomes by receptor-independent routes. RTB-mediated delivery mechanisms are fundamentally different than those used by current ERT drugs. The RTB carrier may therefore provide novel biodistribution and pharmacological behavior to associated ERTs *in vivo*. Evaluation of the RTB:IDUA ERT in the MPS I mouse model^38^,^39^ will be required to determine whether RTB improves ERT access and disease correction in these “hard-to-treat” tissues. If successful in this LSD model, RTB-mediated delivery may have significant implication for therapeutic protein delivery impacting a broad spectrum of diseases.

## Methods

### Construct design and *Agrobacterium*-mediated transient expression

Construction of the cassette containing the double enhanced 35S constitutive plant promoter^40^, a translational enhancer from Tobacco Etch Virus (TEV)^41^, the plant signal peptide derived from the potato patatin tuber storage protein (pat)^42^, and the RTB sequence was described previously (Reidy, 2006, etd-12202006-220049). For the fusion constructs, the RTB sequence was PCR-amplified using *Pfu* DNA polymerase to generate the RTB coding fragment. Sequences encoding human IDUA were synthesized (GeneArt) based on GenBank M74715.1 in two different versions; one utilized the human DNA sequence; the other was codon-optimized based on *Nicotiana tabacum* codon preferences and GeneArt’s expression optimization algorithm. Sequence encoding a C-terminal 6X-histidine tag was added to facilitate detection and purification.

IDUA-His and RTB:IDUA expression cassettes (diagrammed in Fig. 1a) were cloned into the plant expression/transformation vector pBIB-Kan^43^ using In-phusion polymerase dry-down PCR Cloning Kit (Clontech). PCR and In-phusion cloned products were confirmed by DNA sequence analyses.

Plasmids containing the various gene constructs were introduced into *Agrobacterium tumefaciens* strain LBA4404 using a freeze/thaw method^44^. *A. tumefaciens*-mediated transient expression of these constructs in *Nicotiana benthamiana* plants was performed as described previously^13^.

### Extraction and quantification of recombinant protein

Plant tissue was ground in a mortar and pestle under liquid nitrogen and then homogenized with extraction buffer (100 mM Tris-HCl, 150 mM MgCl_2_, 10 mM Na_2_S_2_O_5,_ 2 mM PMSF, and 0.1% Tween 20; pH 7.5). Samples were centrifuged at 13,000 × g for 30 min at 4 °C and the clarified supernatant was used in Western immunoblot analysis, enzymatic activity assays, and lactose binding analysis.

### Western immunoblot analysis

Proteins from crude extracts were fractionated on 12% Novex®NuPAGE Bis-Tris polyacrylamide gel (LifeTechnologies); by SDS-PAGE along with the Dual Color Precision Plus Protein Standard (BioRad). Proteins were transferred to nitrocellulose membrane and immuno-detected using the SNAPi.d. Protein Detection System (EMD Millipore). Membranes were blocked with 3% BSA in PBS + 0.05% Tween-20. A monoclonal anti-IDUA (R&D Systems) was used as the primary antibody and detected using an affinity-purified alkaline phosphatase–conjugated goat anti-mouse secondary antibody (BioRad). Immunoblots were developed with CDP-Star (Roche) chemilluminescent substrate and images captured on film in accordance with manufacturer instructions.

### Enzymatic activity

IDUA enzymatic activity was determined using the fluorogenic substrate 4-methylumbelliferyl-α-iduronide (4MUI) (Santa Cruz Biotechnology). A previously reported protocol for measuring IDUA activity in plant extracts was adapted^9^,^11^. Briefly, 10 μl of substrate solution (0.75 mM 4MUI in assay buffer (0.1 M sodium acetate pH 4.8, 1 mM sodium metabisulphite, 3.5 mg/ml BSA)) was mixed with 5 μl plant crude extract in a black-walled 96-well microtiter plate and incubated in the dark at 37 °C for 60 min. Corresponding extracts from “empty vector” control plants were used to establish background. A 4-methylumbelliferone standard curve was generated by serial dilutions ranging from 0.23–15 μM. Reactions were terminated by adding a pH 10.7 stop solution and fluorescence was detected (excitation = 355 nm, emission = 460 nm). One unit of enzyme activity is defined as 1 nmol of 4-MU produced per min at 37 °C.

### Dual bioactivity assay

An assay was developed to quantify RTB:IDUA fusion proteins that takes into account both RTB and IDUA activities. It is based on lectin-mediated affinity to glycoproteins by RTB and direct IDUA activity using 4MUI. Immulon 4HBX plates were coated with asialofetuin (Sigma-Aldrich) containing solution (300 μg/mL) in bicarbonate buffer (pH 9.5) followed by incubation at room temperature for 1 h. Following a washing step with 0.5% Tween-20 in PBS, 100 μL of diluted samples (1:20) in PBS + 1% BSA were added to the asialofetuin-coated plate and incubated for 1 h at room temperature. Following a second wash step, an aliquot of extracts from plants infiltrated with an empty vector (pBIB-Kan; 5 μl) was added to all test sample wells to control for components that may be present in plant crude extracts. A control crude sample containing an aliquot of the recombinant fusion crude extract (5 μl) was applied to unloaded wells in order to determine the activity emitted from any enzymatically active IDUA (with or without lectin binding activity). Reaction was initiated with 10 μl of substrate solution (0.1 M sodium acetate pH 4.8, 1 mM sodium metabisulphite, 3.5 mg/ml BSA and 0.75 mM 4MUI) and incubated for 60 min at 37 °C. Fluorometric determination for detecting and quantifying fluorescent breakdown of the substrate was performed as described above for IDUA enzymatic assays.

### Protein purification, quantification and confirmation of identity

RTB:IDUA fusion protein and plant-synthesized IDUA control protein were extracted and enriched using affinity chromatography methods; RTB:IDUA was purified based on lectin affinity using lactose resins (Sigma) while the HIS-tagged IDUA (pld-IDUA) was purified by IMAC affinity chromatography using a nickel-based Ni-superflow® resin (Clontech). Tissue was ground in liquid nitrogen and resuspended in 100 mM Tris-HCl, pH 7.5, 150 mM MgCl_2_, 10 mM Na_2_S_2_O_5,_ 2 mM PMSF, and 0.1% Tween 20 at a 1:2 (w/v) ratio. Samples were clarified by centrifugation at 20,000 × g for 20 min. Supernatant of RTB:IDUA-containing extracts were incubated with α-Lactose-agarose resin for 2 h at room temperature prior to transfer to a poly-prep chromatography column (BioRad). Resin was washed with 5X column volumes of 100 mM Tris-HCl buffer, pH 7.5. RTB:IDUA was eluted with wash buffer containing 500 mM D-galactose. For HIS-tagged plant-derived IDUA, the protein was purified on Ni-Superflow® resin as described in the Clontech user manual.

Elution fractions were concentrated using Amicon Ultra-4 centrifugal filters (30,000 MWCO; EMD Millipore). Buffer-exchange with 100 mM Tris-HCl pH 7.0 (for RTB:IDUA) or 50 mM sodium acetate pH 4.8, 500 mM NaCl (for pld-IDUA) was carried out using Zeba^TM^ spin desalting columns, 7,000 MWCO (Thermo). Final fractions were sterile-filtered using a 0.2 μm centrifugal filter. Tissue from plants infiltrated with *A. tumefaciens* containing an empty vector (pBibKan) was extracted and processed following the lactose affinity procedure to serve as a corresponding negative control.

Quantification of the activity units for purified plant-produced products and for mammalian cell-derived mcd-IDUA (R&D Systems) was determined by the IDUA enzymatic activity assay described above. In order to quantify the specific activity and kinetics of RTB:IDUA, the lactose purification fraction was further purified using size exclusion chromatography as described in manufacture manual (Hiload^TM^ 26/60 Superdex^TM^ 200, General Electric).

Purified protein (1 μg) was resolved by SDS-PAGE and Coomassie-stained following manufacturer’s (Thermo) instructions (shown in Supplementary Fig. 1S online). The 120 kDa band was excised and analyzed at University of Arkansas Medical Science Proteomics Core by in-gel trypsin digestion and tandem mass spectrometry (MS/MS) (Supplementary Fig. S2 online).

### Enzyme kinetic analyses

Purified mcd-IDUA (R&D Systems) and RTB:IDUA, (99% and 98% purity, respectively) were assessed under the same assay conditions. RTB:IDUA was further purified using size exclusion chromatography and purity was quantified by BioRad Experion® Pro260 chip (Supplementary Fig. S1 online). Concentration was calculated from A280 measurements and theoretical extinction coefficient of 183,075 ∈ M^−1^ cm^−1^. RTB:IDUA and mcd-IDUA were diluted to 2 nM in assay buffer. Substrate (4MUI) was diluted in assay buffer to concentrations ranging from 2000 to 15.6 μM. Equal volumes (10 μL) of protein and substrate solution were combined to initiate the reaction and incubated as described above. Reactions were terminated by the addition of 280 μl of stop solution. Results were plotted as units of IDUA activity per min for each substrate concentration. Data analysis was performed using GraphPad Prism software.

### Cellular uptake and GAG quantification assay

A normal fibroblast (GM00010) cell line and two diseased cell lines - Hurler fibroblasts (GM01391) and Hurler-Scheie fibroblasts (GM00963) were purchased from Coriell Cell Repositories and cultured in 150 mm plastic dishes in conditions recommended by the provider. For experiments, culture media of confluent cells was replaced with serum-free MEM containing varying test doses of recombinant protein, control protein (purified protein fraction from plants transformed with an empty vector) or no treatment. For receptor inhibition studies, media containing 4 mM mannose-6-phosphate sodium salt or 4 mg/ml mannan (Sigma) was added 2 h prior to addition of various IDUA proteins or control samples. At treatment initiation, proteins stocks were added directly to the inhibitor-containing media and cells were incubated for 24 hours at 37 °C, 5% CO_2_. Following incubation, cells were harvested using Trypsin-EDTA, washed twice with PBS after centrifugation (1000 × g, 4 °C, 5 min), and cell pellets stored at -20 °C for GAG quantification assays.

The glycosaminoglycan (GAG) content in fibroblasts was quantified by measuring the absorbance of the complex formed by sulfated GAGs and dimethylmethylene blue (DMMB) as described elsewhere^45^. Fibroblast responses to the various treatments were analyzed using Dunnett’s multiple comparisons test in GraphPad Prism software.

### High throughput imaging assays

Hurler, Hurler-Scheie, and normal fibroblasts were plated at 10^5^ cells/ml in a black-walled, clear bottom 96-well plate. Following a 3 h period to allow cell attachment, media was replaced with 100 μl serum-free media containing the various test treatments. For M6P receptor inhibition studies, 75 μl of media containing 5.3 mM M6P sodium salt was added 2 h prior to addition of various IDUA variants or control samples; at treatment time, proteins were diluted in 25 μl of media and added to each well yielding a final concentration of 4 mM M6P. Cells were incubated for 24 h at 37 °C and 5% CO_2_.

Following the 24 h treatment period, cells were treated with 600 nM LysoTracker^®^ red (Invitrogen) for 20 min and fixed with 4% paraformaldehyde (8 min). Cells were counterstained with DAPI (Invitrogen) and analyzed using a BD Pathway 855 High Content Bioimager (BD Biosciences).

Images for each well/repetition were taken using a 2 × 2 montage with the 20X objective yielding an average of 200 cells per image. Fluorescent signal (Excitation/Emission) at 560/645 (red) and 380/435 (blue) was acquired using the same exposure and laser autofocus parameters for each well. Image data acquisition was obtained using the Attovision® software analysis tools (BD Biosciences). Cell count was obtained using polygon segmentation for the nucleus blue signal. LysoTracker^®^ regions of interest (ROI) were obtained by polygon segmentation of one of each dots detected at the red signal. All images within an experiment were analyzed using the same image processing and segmentation parameters.

Data analysis was performed using BD Data explorer® software (BD Biosciences). An average of LysoTracker^®^ pixels per cell were calculated for each image. Averages of at least fifteen images (n = 15) were used to estimate the value of each treatment. Plate-to-plate variation was observed depending on the uptake efficiency of the LysoTracker^®^ reagent, stability of the probe or exposure value used for each plate. Due to this inherent variation, untreated disease cells and normal cells were run as controls for each plate. To compare across plates, data was expressed based on the LysoTracker^®^ signal of each treatment as a percentage of the total signal obtained with untreated disease cells. Fibroblast responses to the various treatments were analyzed using Dunnett’s multiple comparisons test in GraphPad Prism software.

## Additional Information

**How to cite this article**: Acosta, W. *et al.* RTB Lectin: a novel receptor-independent delivery system for lysosomal enzyme replacement therapies. *Sci. Rep.* **5**, 14144; doi: 10.1038/srep14144 (2015).

## Supplementary Material

Supplementary Information

## References

[b1] Grabowski G. Treatment perspectives for the lysosomal storage diseases. Expert Opin Emerg Drugs 13, 197–211 (2008). 18321157 10.1517/14728214.13.1.197

[b2] Ratko T., Marbella A., Godfrey S. & Aronson N. Enzyme-Replacement Therapies for Lysosomal Storage Diseases. Agency for Healthcare Research and Quality (US) ; Technical Brief No.: 12(13)-EHC154-EF.(2013). Available at: http://www.ncbi.nlm.nih.gov/books/NBK117219/. (Accessed: 24th November 2014). 23390670

[b3] Sandvig K., Bergan J., Kavaliauskiene S. & Skotland T. Lipid requirements for entry of protein toxins into cells. Prog. Lipid Res. 54C, 1–13 (2014). 24462587 10.1016/j.plipres.2014.01.001

[b4] Sandvig K., Pust S., Skotland T. & van Deurs B. Clathrin-independent endocytosis: mechanisms and function. Curr. Opin. Cell Biol. 23, 413–420 (2011). 21466956 10.1016/j.ceb.2011.03.007

[b5] Sandvig K. & van Deurs B. Membrane traffic exploited by protein toxins. Annu. Rev. Cell Dev. Biol. 18, 1–24 (2002). 12142266 10.1146/annurev.cellbio.18.011502.142107

[b6] Sandvig K. & van Deurs B. Endocytosis and intracellular transport of ricin: recent discoveries. FEBS Lett. 452, 67–70 (1999). 10376680 10.1016/s0014-5793(99)00529-3

[b7] Iversen T. G., Frerker N. & Sandvig K. Uptake of ricinB-quantum dot nanoparticles by a macropinocytosis-like mechanism. J. Nanobiotechnology 10, 1–33 (2012). 22849338 10.1186/1477-3155-10-33 PMC3466139

[b8] Skilleter D. N. & Foxwell B. M. J. Selective uptake of ricin A-chain by hepatic non-parenchymal cells *in vitro*. Fed. Eur. Biochem. Soc. 196, 344–348 (1986). 10.1016/0014-5793(86)80276-9 3949006

[b9] Fu L. H. *et al.* Production and characterization of soluble human lysosomal enzyme α-iduronidase with high activity from culture media of transgenic tobacco BY-2 cells. Plant Sci. 177, 668–675 (2009).

[b10] Radin D. N., Cramer C. L., Oishi K. K. & Weissenborn D. L., inventors; Virginia Tech Intellectual Properties, Inc, Radin D, Cramer C., assignees. Production of lysosomal enzymes in plant-based expression systems. United States Patent 5,929,304.1999 Jul 27.

[b11] Downing W. L. *et al.* Synthesis of enzymatically active human alpha-L-iduronidase in Arabidopsis cgl (complex glycan-deficient) seeds. Plant Biotechnol. J. 4, 169–181 (2006). 17177794 10.1111/j.1467-7652.2005.00166.x

[b12] He X. *et al.* Production of alpha-L-iduronidase in maize for the potential treatment of a human lysosomal storage disease. Nat. Commun. 3, 1–10 (2012). 10.1038/ncomms2070 22990858

[b13] Medrano G. *et al.* Rapid system for evaluating bioproduction capacity of complex pharmaceutical proteins in plants. Methods Mol. Biol. 483, 51–67 (2009). 19183893 10.1007/978-1-59745-407-0_4

[b14] Prince W. S. *et al.* Lipoprotein receptor binding, cellular uptake, and lysosomal delivery of fusions between the receptor-associated protein (RAP) and alpha-L-iduronidase or acid alpha-glucosidase. J. Biol. Chem. 279, 35037–35046 (2004). 15170390 10.1074/jbc.M402630200

[b15] He X. *et al.* Characterization and downstream mannose phosphorylation of human recombinant α-L-iduronidase produced in Arabidopsis complex glycan-deficient (cgl) seeds. Plant Biotechnol. J. 11, 1034–43 (2013). 23898885 10.1111/pbi.12096 PMC4030584

[b16] Gomord V. & Faye L. Posttranslational modification of therapeutic proteins in plants. Curr. Opin. Plant Biol. 7, 171–81 (2004). 15003218 10.1016/j.pbi.2004.01.015

[b17] Shaaltiel Y. *et al.* Production of glucocerebrosidase with terminal mannose glycans for enzyme replacement therapy of Gaucher’s disease using a plant cell system. Plant Biotechnol. J. 5, 579–590 (2007). 17524049 10.1111/j.1467-7652.2007.00263.x

[b18] Unger E. G., Durrant J., Anson D. S. & Hopwood J. J. Recombinant alpha-L-iduronidase: characterization of the purified enzyme and correction of mucopolysaccharidosis type I fibroblasts. Biochem. J. 289, 241–246 (1994). 10.1042/bj3040043 PMC1137449 7998955

[b19] Kim J.-J. P., Olson L. J. & Dahms N. M. Carbohydrate recognition by the mannose-6-phosphate receptors. Curr. Opin. Struct. Biol. 19, 534–42 (2009). 19801188 10.1016/j.sbi.2009.09.002 PMC2771201

[b20] Van Meel E. & Klumperman J. Imaging and imagination: understanding the endo-lysosomal system. Histochem. Cell Biol. 129, 253–66 (2008). 18274773 10.1007/s00418-008-0384-0 PMC2248605

[b21] Urayama A., Grubb J. H., Sly W. S. & Banks W. A. Developmentally regulated mannose 6-phosphate receptor-mediated transport of a lysosomal enzyme across the blood-brain barrier. Proc. Natl. Acad. Sci. USA 101, 12658–12663 (2004). 15314220 10.1073/pnas.0405042101 PMC515112

[b22] Olsnes S. The history of ricin, abrin and related toxins. Toxicon 44, 361–370 (2004). 15302520 10.1016/j.toxicon.2004.05.003

[b23] Lord J. M. & Spooner R. A. Ricin trafficking in plant and mammalian cells. Toxins (Basel). 3, 787–801 (2011). 22069740 10.3390/toxins3070787 PMC3202855

[b24] Hervé F., Ghinea N. & Scherrmann J.-M. CNS delivery via adsorptive transcytosis. AAPS J. 10, 455–72 (2008). 18726697 10.1208/s12248-008-9055-2 PMC2761699

[b25] Broadwell R. D., Balin B. J. & Salcmant M. Transcytotic pathway for blood-borne protein through the blood-brain barrier. Proc. Natl. Acad. Sci. 85, 632–636 (1988). 2448779 10.1073/pnas.85.2.632 PMC279605

[b26] Banks W. & Kastin A. Characterization of lectin-mediated brain uptake of HIV-1 GP120. J. Neurosci. Res. 54, 522–529 (1998). 9822162 10.1002/(SICI)1097-4547(19981115)54:4<522::AID-JNR9>3.0.CO;2-O

[b27] Bies C., Lehr C. M. & Woodley J. F. Lectin-mediated drug targeting: History and applications. Adv. Drug Deliv. Rev. 56, 425–435 (2004). 14969751 10.1016/j.addr.2003.10.030

[b28] Wiley R. G., Talman W. T. & Reis D. J. Ricin transport distinguishes between central and peripheral neurons. Brain Res. 269, 357–60 (1983). 6192871 10.1016/0006-8993(83)90146-4

[b29] Ma L., Hsu C. H., Patterson E., Thadani U. & Robinson C. P. Ricin depresses cardiac function in the rabbit heart. Toxicol. Appl. Pharmacol. 138, 72–6 (1996). 8658515 10.1006/taap.1996.0099

[b30] Wiley R. G. & Kline R. H. IV Neuronal lesioning with axonally transported toxins. J. Neurosci. Methods 103, 73–82 (2000). 11074097 10.1016/s0165-0270(00)00297-1

[b31] Whaley K. J., Hiatt A. & Zeitlin L. Emerging antibody products and Nicotiana manufacturing. Hum. Vaccin. 7, 349–356 (2011). 21358287 10.4161/hv.7.3.14266 PMC3166493

[b32] D’Aoust M. *et al.* The production of hemagglutinin-based virus-like particles in plants: a rapid, efficient and safe response to pandemic influenza. Plant Biotechnol. J. 8, 607–619 (2010). 20199612 10.1111/j.1467-7652.2009.00496.x

[b33] Zimran A. *et al.* Pivotal trial with plant cell-expressed recombinant glucocerebrosidase, taliglucerase alfa, a novel enzyme replacement therapy for Gaucher disease. Blood 118, 5767–5773 (2011). 21900191 10.1182/blood-2011-07-366955

[b34] Pastores G. *et al.* Plant cell-expressed recombinant glucocerebrosidase: Taliglucerase alfa as therapy for Gaucher disease in adults patients previously treated with imiglucerase: 24-month results. Mol Genet Metab 108, S73–S74 (2013).

[b35] Aviezer D. *et al.* A plant-derived recombinant human glucocerebrosidase enzyme—A preclinical and phase I investigation. PLoS One 4, e4792 (2009). 19277123 10.1371/journal.pone.0004792 PMC2652073

[b36] Qiu X. *et al.* Reversion of advanced Ebola virus disease in nonhuman primates with ZMapp. Nature (2014) 10.1038/nature13777. PMC4214273 25171469

[b37] Landry N. *et al.* Preclinical and clinical development of plant-made virus-like particle vaccine against avian H5N1 influenza. PLoS One 5, e15559 (2010). 21203523 10.1371/journal.pone.0015559 PMC3008737

[b38] Clarke L. A. *et al.* Murine mucopolysaccharidosis type I: targeted disruption of the murine alpha-L-iduronidase gene. Hum. Mol. Genet. 6, 503–511 (1997). 9097952 10.1093/hmg/6.4.503

[b39] Ou L., Herzog T., Koniar B. L., Gunther R. & Whitley C. B. High-dose enzyme replacement therapy in murine Hurler syndrome. Mol. Genet. Metab. 111, 116–22 (2014). 24100243 10.1016/j.ymgme.2013.09.008 PMC4014311

[b40] Odell J., Nagy F. & Chua N. Identification of DNA sequences required for activity of the cauliflower mosaic virus 35S promoter. Nature 313, 810 – 812 (1985). 3974711 10.1038/313810a0

[b41] Nicolaisen M., Johansepb E., Poulwp G. B. & Borkhardt B. The 5′ untranslated region from pea seedborne mosaic potyvirus RNA as a translational enhancer in pea and tobacco protoplasts. Fed. Eur. Biochem. Soc. 303, 169–172 (1992). 10.1016/0014-5793(92)80511-e 1607015

[b42] Iturriaga G., Jefferson R. A. & Bevan M. W. Endoplasmic reticulum targeting and glycosylation of hybrid proteins in transgenic tobacco. Plant Cell 1, 381–390 (1989). 2535509 10.1105/tpc.1.3.381 PMC159770

[b43] Becker D. Binary vectors which allow the exchange of plant selectable markers and reporter genes. Nucleic Acids Res. 18, 203 (1990). 2408008 10.1093/nar/18.1.203 PMC330240

[b44] Holsters M. *et al.* Transfection and transformation of Agrobacterium tumefaciens. Mol. Gen. Genet. 163, 181–187 (1978). 355847 10.1007/BF00267408

[b45] Arfi A., Richard M., Gandolphe C. & Scherman D. Storage correction in cells of patients suffering from mucopolysaccharidoses types IIIA and VII after treatment with genistein and other isoflavones. J. Inherit. Metab. Dis. 33, 61–67 (2010). 20084460 10.1007/s10545-009-9029-2

