# Supplementary material for: RTB Lectin: a novel receptor-independent delivery system for lysosomal enzyme replacement therapies
Source: Sci Rep. 2015 Sep 18;5:14144. doi: 10.1038/srep14144 (PMC4585660; doi:10.1038/srep14144)

SUPPLEMENTARY MATERIAL

RTB Lectin: a novel receptor-independent delivery system for lysosomal enzyme replacement therapies

Walter Acosta[1,4], Jorge Ayala[1,3], Maureen C. Dolan[1,2] & Carole L. Cramer [1-3]

[1]Arkansas Biosciences Institute at Arkansas State University-Jonesboro, PO Box 639, State University, AR 72467, USA.

[2]Department of Biological Sciences, Arkansas State University-Jonesboro, State University AR 72467, USA.

[3]BioStrategies LC, PO Box 2428, State University, AR 72467, USA.

[4]Present address: BioStrategies LC, PO Box 2428, State University, AR 72467, USA.

Correspondence should be addressed to C.L.C. (ccramer@astate.edu).

Supplementary Figure S1. RTB:IDUA protein purity. After size exclusion, purified protein (1µg, measured by A_{280}) was size-separated by SDS-PAGE and Coomassie stained following manufacture instructions (Thermo). A single band of 120 kDa was detected (A). Percentage of purity was further confirmed at 98.3% using an automated electrophoresis station BioRad Experion® Pro260 chip following manufacturer's procedures (B).

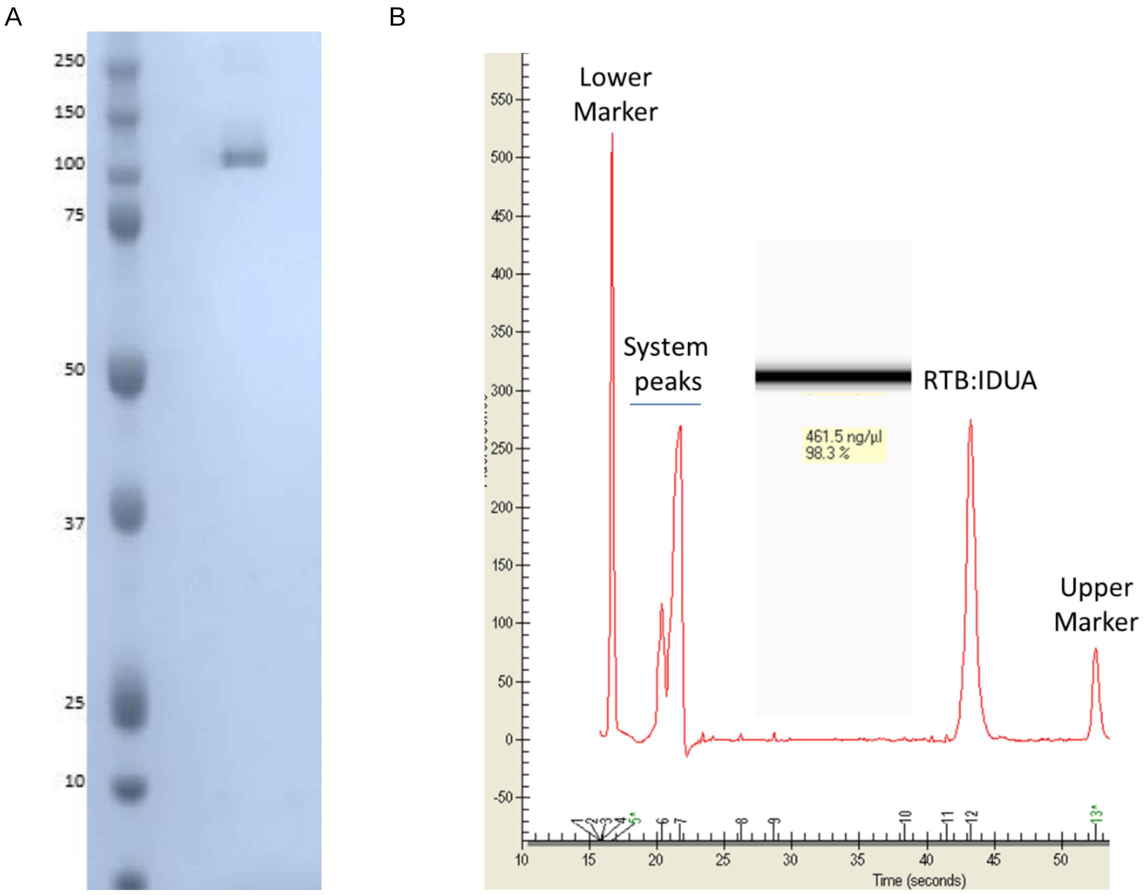

Supplementary Figure S2. RTB:IDUA protein identification. Purified protein detected by Coomassie staining (e.g., Fig. S1) was analyzed at University of Arkansas for Medical Science Proteomics Core by in-gel trypsin digestion and tandem mass spectrometry (MS/MS). Excluding the patatin signal peptide, there is a 37% coverage (334/895) of the sequence encompassing 32 exclusive unique peptides and 46 exclusive unique spectra.

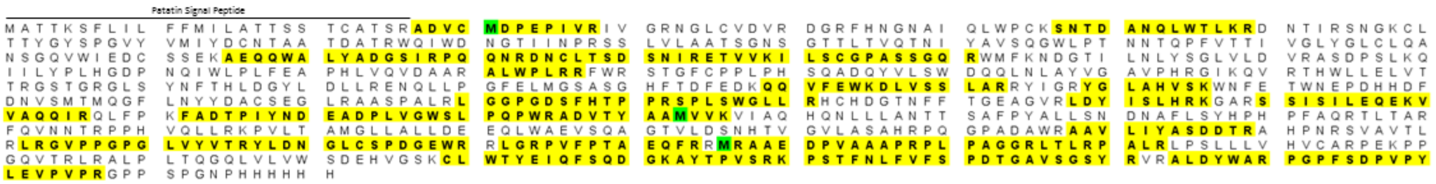

Supplement: Supplementary Information [file srep14144-s1.pdf]
